# Relationship Between Dehydrin Accumulation and Winter Survival in Winter Wheat and Barley Grown in the Field

**DOI:** 10.3389/fpls.2019.00007

**Published:** 2019-01-29

**Authors:** Pavel Vítámvás, Klára Kosová, Jana Musilová, Ludmila Holková, Pavel Mařík, Pavlína Smutná, Miroslav Klíma, Ilja Tom Prášil

**Affiliations:** ^1^Department of Genetics and Plant Breeding, Crop Research Institute, Prague, Czechia; ^2^Department of Crop Science, Breeding and Plant Medicine, Faculty of AgriSciences, Mendel University, Brno, Czechia; ^3^Research Centre SELTON, Ltd., Stupice, Czechia

**Keywords:** winter survival, vernalization, apical development, field trials, dehydrins, winter wheat, winter barley

## Abstract

Low temperatures represent a crucial environmental factor determining winter survival (WS) of barley and wheat winter-type varieties. In laboratory experiments, low temperatures induce an active plant acclimation response, which is associated with an enhanced accumulation of several stress-inducible proteins including dehydrins. Here, dehydrin accumulations in sampled wheat (WCS120 protein family, or WCS120 and WDHN13 transcripts) and barley (DHN5 protein) varieties grown in two locations for two winters were compared with the variety WS evaluated by a provocation wooden-box test. A high correlation between dehydrin transcripts or protein relative accumulation and variety WS score was found only in samples taken prior vernalization fulfillment, when high tolerant varieties accumulated dehydrins earlier and to higher level than less tolerant varieties, and the plants have not yet been vernalized. After vernalization fulfillment, the correlation was weak, and the apical development indicated that plants reached double ridge (DR) in barley or stayed before DR in wheat. Dehydrin proteins and transcripts can be thus used as reliable markers of wheat or barley variety winter hardiness in the field conditions; however, only at the beginning of winter, when the plants have not yet finished vernalization. In wheat, a higher correlation was obtained for the total amount of dehydrins than for the individual dehydrin proteins.

**HIGHLIGHTS:**

- More tolerant winter-type wheat and barley plants reveal higher threshold induction temperatures for dehydrin accumulation in comparison to less tolerant varieties. Thus, more tolerant winter cereals have higher dehydrin levels than the less tolerant ones upon the same ambient temperature in November samplings.
- A significant correlation between dehydrin transcript/protein accumulation and winter survival was found in both winter wheat and winter barley plants in the field conditions, but only prior to vernalization fulfillment.

## Introduction

Low temperatures, such as cold and frost, significantly affect overwintering of winter cereals. Winter cereals respond to low temperatures via two processes: cold acclimation and vernalization. Cold acclimation means a complex of processes aimed at enhancement freezing tolerance to enhance plant survival under adverse low temperatures. Vernalization means a developmental adaptation to regular long-term periods of low temperatures in temperate climates. Epigenetic modifications of the genes involved in flowering pathway prevent a premature transition from vegetative into reproductive stage before the periods of long-term low temperatures during winter. Vernalization means an acquisition or an acceleration of flowering via a long-term chilling treatment (Chouard, 1960) and this process is associated with complex developmental (epigenetic) modifications enabling activation of flowering pathways genes. It is well known that vernalization fulfillment in winter cereals leads to decreased ability of the plants to induce enhanced freezing tolerance during cold reacclimation treatment; however, molecular mechanisms underlying this phenomenon still remain largely unknown (Kosová et al., 2008; Vitámvás and Prášil, 2008; Dhillon et al., 2010).

The dynamics of freezing temperatures during winter affects the ability to acquire enhanced frost tolerance in winter cereals. Molecular studies have shown that fulfillment of vernalization requirement followed by an enhanced expression of *VRN1* gene leads to an upregulation of *FT1* homolog and other genes involved in flowering pathway while a downregulation of cold-inducible *CBF* pathway including downstream *COR/LEA* genes although precise mechanisms still remain unknown (Dhillon et al., 2010; Deng et al., 2015). Vernalization fulfillment is also regulated by photoperiod; it was found out that daylength prolongation leads to induction of FT1/VRN3 gene which acts as a positive inducer of VRN1 gene (Yan et al., 2006). Vernalization thus leads to a reduced plant ability to newly induce enhanced freezing tolerance after a period of relatively high temperatures leading to plant deacclimation. Similarly, our previous study on cold acclimation, deacclimation and reacclimation in frost-tolerant winter wheat Mironovskaya 808 grown in controlled conditions revealed a significant decrease in the ability to establish enhanced frost tolerance determined as lethal temperature for 50% of the sample (LT50) values in vernalized winter wheat plants subjected to cold reacclimation when compared to unvernalized ones (Vitámvás and Prášil, 2008). Dehydrins represent an important group of LEA-II proteins induced by several stress factors including low temperatures via both ABA-dependent as well as ABA-independent (CBF) signaling pathways (Kosová et al., 2007; Battaglia et al., 2008). Previous studies on cold-treated winter wheat and barley plants grown in controlled environment have revealed correlations between dehydrin transcript and protein accumulation and acquired frost tolerance in fully cold-acclimated plants (Kosová et al., 2008, 2010; Holková et al., 2009; Vítámvás et al., 2010) as well as in plants grown under mild temperatures (Vítámvás et al., 2010; Kosová et al., 2013). However, no analogous data were presented regarding field-grown plants which are subjected to changing temperatures.

Global climate change could lead to deacclimation of vernalized winter cereals during later winter phases (January, February) followed by freezing temperatures which can result in serious plant frost damage. Dehydrin accumulation at transcript and protein levels can help the researchers and the breeders to assess plant sensitivity to frost damage at the given plant developmental stage not only under controlled conditions of growth chambers, but also under field conditions. Recently, several studies were published on cold-inducible dehydrin accumulation in wheat and barley plants grown under controlled conditions in growth chambers (Houde et al., 1992a,b; Danyluk et al., 1994, 1998; Vágújfalvi et al., 2000, 2003; Crosatti et al., 2003; Kobayashi et al., 2005; Vítámvás et al., 2007, 2010; Kosová et al., 2008, 2013). However, for determination of the possibility to utilize dehydrin transcript or protein relative accumulation as a marker of plant winter hardiness, field experiments are necessary. Currently, only a few studies are available on COR/LEA transcript or protein accumulation under the field conditions (Giorni et al., 1999; Crosatti et al., 2008; Ganeshan et al., 2009; Pomortsev et al., 2017). Their results reveal a great variability in COR/LEA transcript or protein levels throughout different winter seasons as well as throughout a single winter season due to a large variability in field growth conditions. A positive relationship between an accumulation of COR14 (chloroplast-located LEA-III protein) and winter survival (WS) capacity was found in a set of 10 barley varieties of both winter and spring growth habits grown in the field conditions in Italy and sampled in November and December revealing higher COR14 accumulation in the winter varieties with respect to the spring ones (Giorni et al., 1999). In contrast, Crosatti et al. (2008) showed only a relationship between plant growth stage and their WS score, but no relationship between plant WS and COR14b accumulation in winter and spring barley varieties grown in the field when sampled in January and February.

Therefore, the aim of our study was to evaluate dehydrin relative accumulation at both transcript and protein levels in wheat and barley varieties grown under field conditions at Crop Research Institute (CRI), Prague, and at Selgen, Lužany, during winters in 2013/14 and 2014/15. Furthermore, one of the major aims was to study the dynamics of dehydrin relative accumulation and plant vernalization fulfillment (non-vernalized vs. vernalized plants) during winter. Moreover, the possibility of distinguish cereal varieties with different level of WS (frost tolerance) by dehydrin analysis of crowns sampled from field was tested and discussed.

## Materials and Methods

### Plant Materials

Winter cereals were cultivated under field condition at CRI, Prague (50°5′5″N, 14°17′58″E; wheat and barley) and Selgen, a.s. – Lužany (49°32′40″N, 13°18′21″E; barley), Czechia during winters 2013/14 and/or 2014/15. During the 2013/14 winter, winter barley varieties Lancelot, Fridericus, Saffron, Fabian, Kaylin were grown in Prague and Lužany. In Prague, wheat varieties Bohemia, Dulina, Sultan and Aranka were cultivated during 2013/14 winter and Bohemia, Gordian, Cimrmanova Raná, Elan, Dagmar, Julie, Tobak and Turandot were cultivated during 2014/15 winter. During the winter 2014/15, barley varieties Lancelot, Fridericus, KWS Meridian, Sylva, Saffron were grown in both locations and Fabian, Lester and Travira only in the location Lužany. All seeds used in the experiments were obtained from Central Institute for Testing and Supervising in Agriculture (Brno, CZ). During sampling (CRI – 5.12.2013, 29.1.2014, 24.11.2014, 19.1.2015; Lužany – 8.1.2014, 9.12.2014), crowns from 10 plants were taken in triplicates directly in the field and the samples were immediately frozen in liquid nitrogen.

Winter-survival of the varieties was assessed by a provocation wooden-box test under natural conditions that was described in detail in the paper by Prášil and Rogalewicz (1989). In short, plants were grown in wooden boxes (40 cm × 30 cm × 15 cm) filled with field soil and placed at two heights above the ground (5 and 50 cm) during winter. Four biological replicates containing a set of 32 plants per each replicate were employed for the test. In spring, plant survival was assessed and average WS was calculated for each variety from multi-year trials according to Prášil et al. (1989).

Frost tolerance test: In order to determine if cereal plants are sufficiently frost tolerant at the beginning of winter, the wheat and barley varieties taken from the field at first sampling (5.12.2013) were subjected to a direct freezing test in laboratory freezers according to Prášil et al. (Prášil et al., 2007). Four different freezing temperatures and 20 plants per each freezing temperature were used for the freezing test. Then, after 3 weeks of plant regeneration, the lethal temperature (LT50) was calculated based on the model of Janáček and Prášil (1991).

Apical development was determined from changes in the morphology of the shoot apex (Prášil et al., 2004). Shoot apices from three plants were dissected and further investigated under microscope (Wilde, Germany). The decimal code of the apex was scored for the early development stages as follows (Nátrová and Jokeš, 1993): 11 – Early vegetative development of the shot apex; 13 – Beginning of the shoot apex elongation; 16 – Beginning of single ridges (leaf primordia); 19 – Single ridges along the whole shoot apex, 20–24 Formation of double ridges (DRs) (from early to late stage).

Days to heading, indicating the time of vernalization saturation, were determined for 15 plants taken at the sampling day (Prášil et al., 2004). The plants were grown in a field soil in a glasshouse at 20 ± 2°C and a 16-h photoperiod provided by supplemental lighting (high intensity discharge lamps LU/400/T/40, Tungsram, Hungary). The heading time for each variety was expressed as the number of days from sampling until the 50% of plants presented the complete spike outside the sheath of the flag leaf in the main shoot.

### Transcript Analysis

Total RNA was isolated from 100 mg crown tissues using RNase Plant Mini Kit (Qiagen, Hilden, Germany). RNA purification was performed using Turbo DNase kit (Ambion, United States). The first cDNA chain was synthesized from 1 μg of purified total RNA using QuantiTect^®^ Reverse Transcription kit (Qiagen, Hilden, Germany) and qPCR were performed with QuantiTect^®^ SYBR^®^ Green PCR Kit (Qiagen, Hilden, Germany). The following qPCR conditions were applied: Starting with 95°C for 15 min followed by 40 cycles at 94°C for 15 s, 60°C for 30 s, and 72°C for 30 s. *Ubiquitin1* was used as an internal reference gene for both *WCS120* and *WDHN13*. The following primers were used: *WCS120* F: TTCACGGACAACAGTGTG and R: CTGCGTCTGTCTCTTGGATAAG; *WDHN13* F: GCACGGTGACCACCAGCAC and R: TAGCGGGTCGGGCGCGGGC; *Ubiquitin1* F: GCATGCAGATATTTGTGAA and R: GCAGCTTACTGGCCAA.

Further details on *WCS120* PCR conditions are given in Ganeshan et al. (2009) and *WDHN13* PCR conditions are given in Solařová et al. (2016).

Gene expression was evaluated as normalized relative gene expression (NRE) calculated according to Pfaffl (2001).

The values of gene expression were normalized versus expression of ubiquitin gene and relative to the value of internal calibrator, i.e., average value of tested gene expression in leaves of variety Bohemia in November sampling. The expression level of tested genes in leaves of this variety was taken as the standard 1 (100%). Three independent (2 ×± SD) sample measurements were averaged.

### Accumulation of Dehydrin Proteins

The level of dehydrins was studied by immunoblotting of protein soluble upon boiling with anti-dehydrin antibody (Enzo Life Sciences) described by Vítámvás et al. (2010). In short, proteins soluble upon boiling were extracted from crown tissues by Tris buffer (0.1 M Tris-HCl, pH 8.8 containing Complete EDTA-free protease inhibitor cocktail (Roche) from frozen crowns ground to fine powder under liquid nitrogen using mortar and pestle. After boiling step (15 min), proteins were precipitated under acetone with 1% ß-mercaptoethanol. The protein concentration was determined according to 2-D Quant kit manual (GE-Healthcare). About 3 μg of extracted proteins of boiling-soluble fraction were loaded into each well of 10% SDS-PAGE (Laemmli, 1970). The proteins were electrophoretically transferred to nitrocellulose (0.45 μm; Pharmacia Biotech). The anti-dehydrin antibody bound to protein bands were visualized by BCIP/NBT staining (Bio-Rad). GS-800 calibrated densitometer (Bio-Rad) was used for image capture of the visualized dehydrin bands. DHN5 protein in barley samples was determined by its specific position on the immunoblots given by its molecular weight (58.5 kDa, apparent molecular weight of 86 kDa on SDS-PAGE) which is much higher than molecular weights of the remaining barley dehydrin proteins (Tommasini et al., 2008). Densitometric quantification of the dehydrin bands was done using Quantity One version 4.6.2 software (Bio-Rad). Density values from wheat cv Bohemia and barley Lancelot, Fridericus and Saffron (i.e., cvs analyzed in all sampling) were used for normalization of densities between all gels.

### Statistical Analysis

Statistical evaluation of plant morphological and developmental characteristics (shoot apex development, days to heading) was carried out using ANOVA analysis followed by Duncan’s multiple range test (significance at 0.05 level). Statistical evaluation of dehydrin transcript and protein relative accumulation was carried out using ANOVA analysis followed by a multiple range test (LSD at the 0.05 level) using averages calculated from three repetitions. Correlation analysis was carried out using Pearson’s correlation coefficient (R) and significance was considered at 0.05 level. All analyses were performed using Unistat software (*Unistat version 5.1, Unistat Ltd.*, London, United Kingdom).

## Results

### Winter Survival and Frost Tolerance of Tested Varieties

**FIGURE 1:**
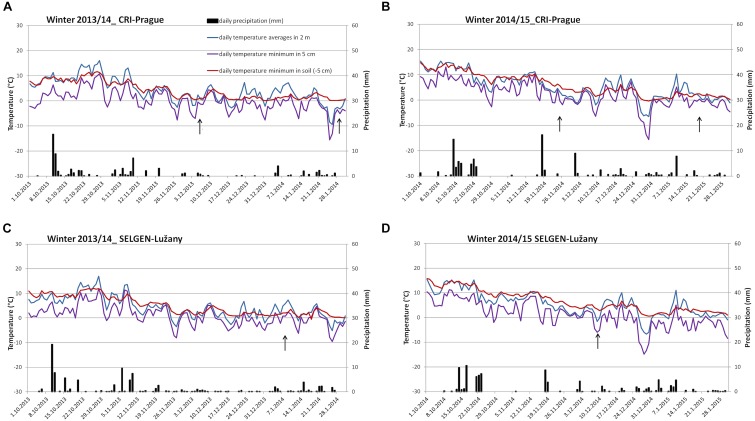
Temperature and precipitation during winters 2013–2015 at two locations. **(A)** Winter 2013/14 in CRI- Prague, **(B)** winter 2014/15 in CRI- Prague, **(C)** winter 2013/14 in Selgen-Lužany, and **(D)** winter 2014/15 in Selgen-Lužany. Arrow indicates date of the sampling.

During winters 2013/14 and 2014/15, the longer periods of higher temperature above 5°C were experienced by winter wheat and barley plants on both field locations during December and January (Figure 1). However, the air temperature decreased gradually during autumn, so the plants reached sufficient frost resistance at the beginning of December 2013 (Supplementary Table S1). Due to higher minimal temperature of soil, the cereals had no mortality in the field during these winters and the provocation wooden-box trials were necessary to distinguish differences between tested varieties (Table 1). The winter wheat varieties had WS from 70 (Bohemia with the highest hardiness) to 30 (Elan with the lowest hardiness). WS of barley varieties ranged from 60 (Lester with the highest hardiness) to 19 (Saffron - the lowest hardiness). In addition, one spring wheat Aranka (WS 4) was added to winter wheats to compare dehydrin relative accumulation in genotypes with and without vernalization requirement.

**Table 1:** Wheat and barley varieties used in experiments and their winter survival (WS in %) evaluated by a provocation wooden-box test under natural conditions.

Wheat	WS	Barley	WS
Bohemia	70	Lester	59
Dulina	64	Fridericus	58
Julie	56	Fabian	58
Cimrmanova raná	55	Kaylin	55
Turandot	53	Lancelot	46
Dagmar	51	KWS Meridian	43
Gordian	40	Travira	40
Tobak	38	Sylva	29
Sultan	33	Saffron	19
Elan	30		
Aranka	4		


*All varieties (except for Aranka) are winter*.

### Apex Development and Fulfillment of Vernalization

**FIGURE 2:**
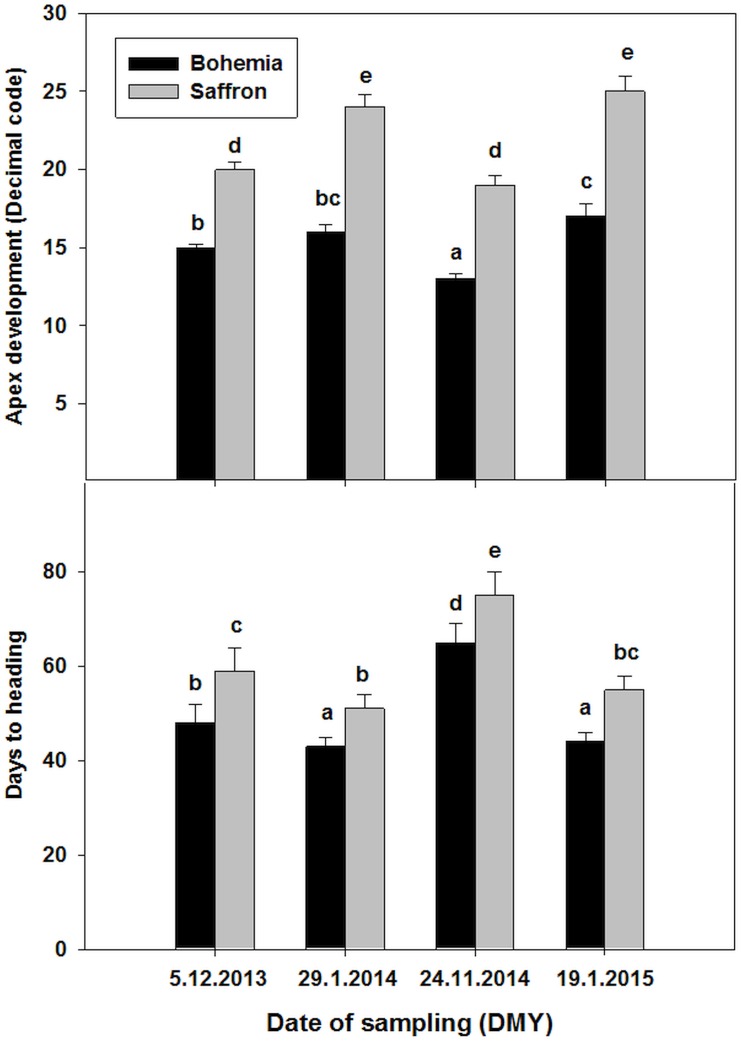
Development of shoot apex and days to heading evaluated in greenhouse for wheat Bohemia and barley Saffron sampled from field during two winters (2013/2014 and 20104/2015). Error bars indicate SD, different letters indicate significant differences at 0.05 level using ANOVA analysis followed by Duncan’s multiple range test. Decimal code 20 indicates the double ridge stage (see more in the methods).

The tests for apex development and vernalization fulfillment were carried out only on plants taken in CRI – Prague, and for wheat Bohemia and barley Saffron varieties because they were performed for both winter seasons (Figure 2). The barley variety reached the DR stage (value 20 and more) in December and held it during January while the wheat did not reach the DRs stages and stayed at the beginning of the shoot apex elongation with single ridges (leaf primordia) minimum till the end of January. Nevertheless, both wheat and barley varieties were at an early stage of apex development in the December sampling day than in the January sampling days.

Similarly, the days to heading for the taken plants growing in the glasshouse were significantly longer for barley or wheat plants sampled at December than later (Figure 2). On the other hand, the differences between days to heading in plants sampled in January in both winter seasons were not significant for the wheat or barley indicating that vernalization fulfillment was accomplished during December for both wheat and barley varieties.

### Dehydrin Protein Relative Accumulation

**FIGURE 3:**
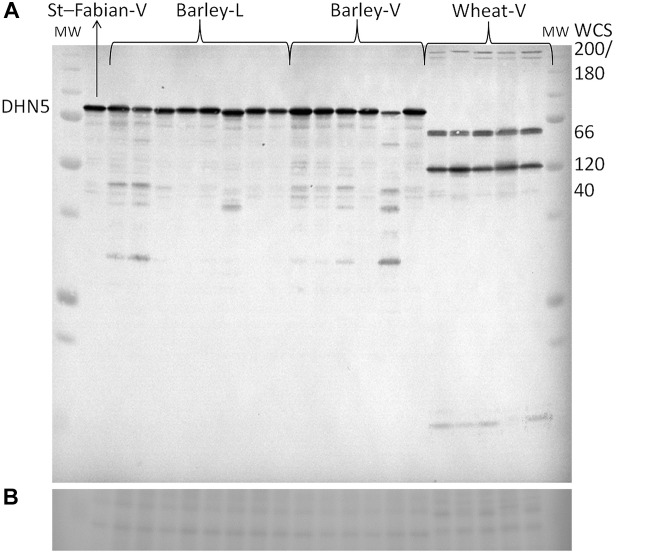
Representative visualization of **(A)** immunoblot of barley and wheat dehydrins extracted from crowns sampled on January 2014. **(B)** The visualization of sample load (cut of the same membrane stained by Ponceau S). Order of the samples from left to right – St-Fabian-V – mixture from all three biological replicates, Kaylin 2nd biological replicate, Kaylin, Travira, Fabian, Lancelot, Fridericus, Saffron, Sylva, Lancelot, Saffron, Kaylin, Fabian, Fridericus, Saffron 2nd biological replicate, Bohemia, Sultan, Dulina, Aranka, Bohemia 2nd biological replicate. Abbreviations: L – samples from SELGEN, Lužany; MW – ALL Blue Precision Plus Protein Standards (Bio-Rad); St – standard for gel normalization of quantity; V – samples from CRI, Prague.

All tested cereal varieties accumulated dehydrins (WCS200, WCS180, WCS66, WCS120, WCS40 in wheat and DHN5 in barley) in the field at all sampling dates (Figure 3 and Supplementary Figure S1). The dynamics of dehydrin protein relative accumulation revealed a variability in protein accumulation during winter seasons (November, December, January; Supplementary Figure S2). The high tolerant varieties (higher WS) accumulated dehydrins to higher levels and much earlier than less frost tolerant varieties. In November and December, the highest level of wheat dehydrin accumulation was found in Bohemia and the lowest in Aranka. However, in January, the highest and lowest wheat dehydrin accumulation was found in middle tolerant varieties Turandot and Tobak, respectively. In barley, the highest level of DHN5 was found in Fridericus and the lowest in Saffron in crowns sampled in November. However, middle frost tolerant variety Lancelot accumulated the highest level of DHN5 in December and November. In December and November, the lowest level of DHN5 was detected in KWS Meridan and Sylva, respectively (Supplementary Figure S1). Thus, differences in dehydrin levels between wheat or barley varieties declined during the winter (December and January) as less tolerant varieties gradually increased dehydrin accumulation to the level of the high tolerant varieties. Therefore, the highest dehydrin accumulation was found in plants sampled during January. Interestingly, sensitive spring variety Aranka showed high variability in samples from January.

### Relationship Between Winter Survival and Wheat and Barley Dehydrin Proteins

**FIGURE 4:**
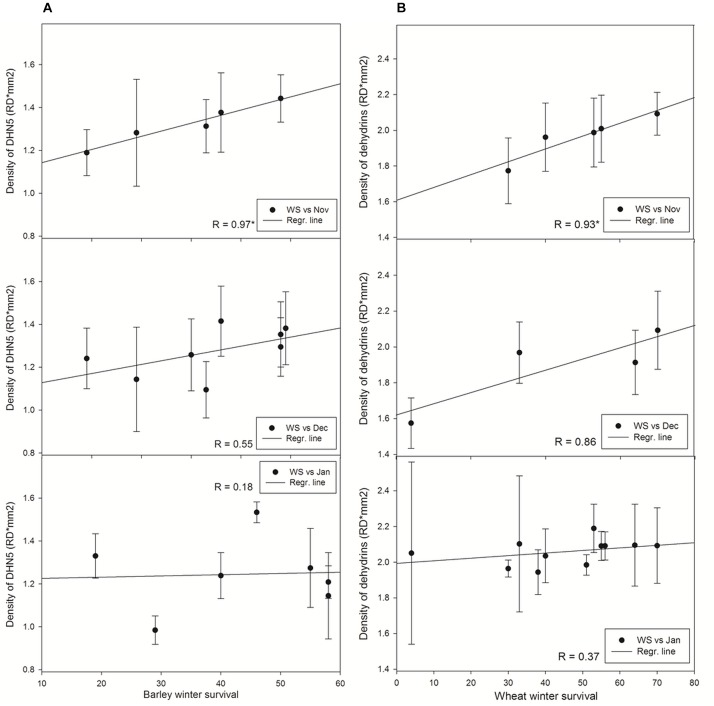
Relationships between winter survival and **(A)** accumulation of DHN5 in barley, **(B)** accumulation of dehydrins in wheat. Asterisk marks significant correlation coefficient R (*P* ≤ 0.05). Dec, December samplings; Jan, January samplings; Nov, November; WS, winter survival.

In wheat, the level of WCS120 alone was not significantly correlated with WS of the tested varieties in any sampling. However, the total amount of the most abundant wheat dehydrins (WCS200, WCS180, WCS120, WCS66, and WCS40; Figure 3) extracted from plants sampled in November 2014 significantly correlated with WS of varieties (Figure 4 and Supplementary Table S2). We could apply this approach due to similarities in the patterns of dehydrin proteins between the studied wheat varieties. Moreover, the total amount of dehydrins from all samples together correlated with the WS of plants (*R* = 0.80; *n* = 11; *P* ≤ 0.01). However, the lower correlation values were found in the plants sampled later. Removal of spring wheat variety Aranka from December and January samplings did not significantly change the correlation (December: *R* = 0.747; *n* = 3; *P* = 0.463; January: *R* = 0.511; *n* = 10; *P* = 0.131; sum: *R* = 0,678; *n* = 10; *P* ≤ 0.05).

In barley varieties, where the most abundant dehydrin protein is DNH5 under cold treatments (Figure 3), the dehydrin showed the same trend in accumulation as dehydrins in wheat varieties. A significant correlation with WS as found only in dehydrins extracted from barley varieties sampled on November 2014. The later sampling, the lower correlation coefficient was revealed (Figure 4). Moreover, the correlation of mean DHN5 levels calculated from all samplings was not significant (*R* = 0.46, *n* = 9).

Interestingly, the lowest correlation coefficient was revealed in the latest samplings of barley and wheat varieties contrary to extended set of varieties (9 and 11, respectively; Figure 4) in the samplings.

### Dehydrin Transcript Relative Abundance in Wheats

The NRE of *WCS120* and *WDHN13* genes were evaluated in the crown tissues of 5 winter wheat varieties at the beginning of November 2014 and then at the end of January 2015 in extended set of varieties (8 varieties) (Supplementary Figure S3). In both terms, variety dependent differences in the NRE level of the two tested genes were observed, but the results reflected significant variability within varieties. In November, the highest expression level of both genes was detected in the Bohemia variety and the lowest in the Gordian variety. The difference was larger than 50% of the NRE *WDHN13* gene and 20% for the NRE of the *WCS120* gene. Overall, the level of expression of both genes under observation slightly increased in January. However, the results were not unambiguous in this respect. While in some varieties an increase in NRE of these genes was observed compared to the first term (Cimrmanova raná, Gordian) in others, decreased levels of expression (Turandot/*WDHN13*, Bohemia/*WCS120*) were detected.

### Winter Survival and Level of Wheat Dehydrin Transcripts

**FIGURE 5:**
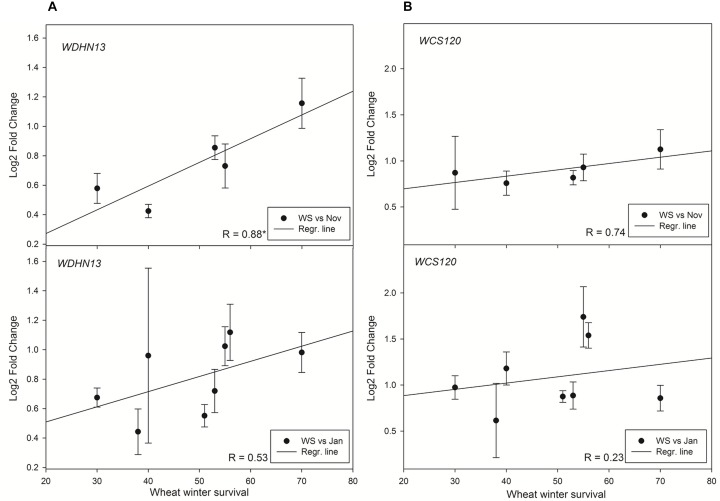
Relationships between WS and **(A)** normalized relative gene expression of *WDHN13* transcript and **(B)** normalized relative gene expression of *WCS120* transcript in wheat. Asterisk marks significant correlation coefficient R (*P* ≤ 0.05). Abbreviations: Jan, January samplings; Nov, November samplings; WS, winter survival.

The results obtained on dehydrin proteins were also validated at transcript level. Interestingly, *WCS120* transcript did not correlate with WS in any sampling (Figure 5). However, the level of transcript of low molecular weight dehydrin gene *WDHN13* revealed a significant correlation with WS in plants sampled on November (2014). No significant correlation was found in later sampling (January 2015).

Unlike DHN5 protein, *Dhn5* transcript levels were not determined in field-grown winter barleys since no significant correlation between *Dhn5* transcript levels and plant acquired frost tolerance determined as LT50 was found in young barley plants grown under regulated conditions (Kosová et al., 2008).

## Discussion

Low temperatures induce cold acclimation processing including profound alterations in proteome composition in winter cereals leading to enhanced acquired frost tolerance. In winter cereals, cold acclimation process is associated with an enhanced accumulation of dehydrin proteins, namely Kn-type dehydrins including WCS120 proteins in wheat and DHN5 protein in barley (Houde et al., 1992b; Sarhan et al., 1997; Vítámvás et al., 2007; Kosová et al., 2008).

Vernalization is a developmental adaptation preventing a premature transition to cold (frost)-susceptible reproductive stage which is ensured by repressing flowering-promoting genes at epigenetic level (Sung and Amasino, 2004). In winter cereals, fulfillment of vernalization requirement is associated with an induction of *VRN-1* gene expression which reveals a negative effect on CBF/COR pathway (Dhillon et al., 2010). Thus, vernalization leads to a decrease in plant ability to induce frost tolerance following a deacclimation period leading to COR protein degradation. In harsh winter with continuous freezing temperatures which are typical for regions with continental climate such as Canada or Siberia, winter cereals maintain high levels of freezing tolerance and freezing tolerance-associated proteins such as dehydrins throughout the whole winter due to continuous freezing temperatures (no deacclimation) (Pomortsev et al., 2017).

However, in mild winters, periods of freezing temperatures are followed by thawing and above-zero temperatures leading to plant deacclimation associated with degradation of cold-responsive proteins such as dehydrins. As a consequence, vernalized winter cereals fail to accumulate enhanced levels of dehydrin proteins when exposed to freezing temperatures following periods of above-zero temperatures associated with deacclimation processes (Vitámvás and Prášil, 2008). Thus, the highest dehydrin accumulation in middle frost tolerant varieties observed in wheat and barley crowns sampled in January could be explained by combined effect of deacclimation processes during observed mild winters (Figure 1) and saturation of vernalization requirements (Figure 2). Under these processes, different varieties could react with different rates of accumulation and degradation of dehydrins that is not closely related to their WS. Comparison of the wheat sets with or without spring wheat Aranka sampled in January revealed that wheats expressing *VRN1* gene are susceptible to deacclimation periods.

Similarly, Pomortsev et al. (2017) also showed changes in dehydrin profiles in winter wheat cv. Irkutskaya at different samplings during winter season in Central Siberia, Irkutsk, Russia (November, February, March). The authors detected the presence of 29 kDa dehydrin protein band in winter wheat only in autumn samplings (November) while it was absent in winter and spring samplings (February, March). Ganeshan et al. (2009) also showed a decrease in Cor/Lea transcripts throughout winter season in Norstar-Manitou set of substitution lines except for winter Manitou *Wcor14* transcript accumulation which continued to increase throughout winter. Moreover, Ganeshan et al. (2009) found fluctuations in transcript levels of selected *Cor/Lea* genes (*WCS120*, *Wcor410*, *Wcor14*) throughout different winter seasons. The authors interpreted the fluctuations in *Cor/Lea* transcript levels between the different winter seasons as reflections of soil temperature fluctuations.

Comparison at transcript level revealed that the most abundant cold-inducible dehydrin in wheat was *WDHN13* (K2) while at protein level, the most abundant cold-inducible dehydrin was WCS120 (K6). The difference can be explained by the fact that dehydrin transcripts are detected using specific primers against unique sequences while dehydrin protein primary antibodies were raised against dehydrin conserved sequence of K-segment. Therefore, dehydrin proteins of Kn type with multiple K segments in their molecules (DHN5 - K9; WCS120 - K6) are overrepresented on the immunoblots when compared to dehydrins with a lower copy number of K segments. Nevertheless, when similar patterns of the individual dehydrin proteins with different copy numbers of K segments are found in studied varieties the total amounts of dehydrin proteins can be compared with the same reliability as individual dehydrins. The significant correlation of *WDHN13* transcript contrary to non-significant correlation of *WCS120* transcript with WS revealed the importance of the comparison expression of more gene family members to adapt proteome results on expression level.

A significant correlation between dehydrin protein relative accumulation (DHN5 in winter barleys; total amount of WCS120 proteins in winter wheats) and plant WS scores was found in early samplings (November). It has been shown in our previous studies that relative abundance of cold-inducible Kn-type dehydrin proteins in cold-acclimated non-vernalized barley (Kosová et al., 2008) and winter wheats (Vítámvás et al., 2007) as well as dehydrin transcripts (*WCS120*, *WDHN13*) in winter wheats (Holková et al., 2009) reveals a correlation to plant frost tolerance determined as LT50 or plant WS. However, these data were obtained only on young plants in vegetative phase (a 3-leaf stage) grown under controlled conditions of growth chamber where all environmental factors can be strictly controlled.

Results achieved by Giorni et al. (1999) and Crosatti et al. (2008) regarding COR14 (not belongs to dehydrins) accumulation in spring and winter barley in the field conditions in Italy support our data. A positive relationship between COR14 accumulation and plant WS was found when sampled in November and December (Giorni et al., 1999) while no significant relationship was found between COR14b accumulation and WS when sampled in January and February (Crosatti et al., 2008). Similarly, Rizza et al. (2011) found no significant relationship between COR14 accumulation and frost tolerance in a set of European barley cultivars when subjected to 1 or 2 weeks of cold acclimation at 3/1°C (day/night).

Results of immunoblot analyses revealed that unlike controlled conditions in growth chamber where a significant correlation between a single WCS120 protein (50 kDa) and wheat acquired frost tolerance was found, a total amount of all WCS120 proteins detected in the immunoblots has to be taken to obtain a significant correlation to WS in winter wheat plants grown in the field. This finding indicate that winter hardiness/frost tolerance represents a quantitative trait of cumulative nature whose resulting level represents a result of additive effects of several differential factors including an accumulation of stress-protective proteins (Thomashow, 1999; Ruelland et al., 2009). It is more probable that winter hardiness which is determined by several stress-protective factors such as accumulation of stress-protective proteins and osmolytes correlates rather with more complex characteristics such as an accumulation level of a total amount of cold-inducible dehydrin proteins rather than a single dehydrin protein.

Studies aimed at an induction of freezing tolerance (LT50) or dehydrin proteins (transcripts) accumulating under a series of decreasing temperatures indicate that the highly frost-tolerant winter varieties of Triticeae start inducing frost tolerance (LT50) and accumulating dehydrin proteins at higher growth temperatures with respect to less tolerant ones (Fowler, 2008; Vítámvás et al., 2010; Kosová et al., 2013). Therefore, in autumn, the highly frost-tolerant varieties start accumulating dehydrin proteins at higher temperatures, i.e., earlier, with respect to the less tolerant ones. Therefore, we observed relatively high differences in dehydrin levels between the differently frost-tolerant winter varieties in November. At later sampling dates following longer periods of low temperatures, also the less frost-tolerant winter wheat and barley varieties start accumulating enhanced dehydrin proteins thus the differences in dehydrin protein relative accumulation between highly frost-tolerant winter varieties with respect to the less tolerant ones became relatively lower when compared to the earlier sampling dates. This concept is analogous to dehydrin samplings from plants grown under different temperature regimes since in both cases, the sum of growth temperatures multiplied by their duration, i.e., the number of degree-days, is crucial for an induction of dehydrin protein relative accumulation in a given winter cereal variety. Thus, it can be concluded that highly frost-tolerant winter cereals (such as barley Fridericus or wheat Bohemia) require lower total values of degree-days for an induction of enhanced dehydrin protein accumulation when compared to less frost-tolerant ones.

It is well-known from experimental data that vernalization fulfillment in winter-type cereals (winter wheat, winter barley) significantly decreases their ability to induce enhanced frost tolerance under cold acclimation treatment. Recently, it was found out that vernalization-induced expression of *VRN-1* gene leads to a down-regulation of cold-inducible CBF transcription regulators and their downstream genes including cold-inducible dehydrins (Dhillon et al., 2010). Under a continuous cold treatment, vernalized winter cereals retain enhanced levels of cold-inducible COR proteins including dehydrins due to their previous accumulation prior to vernalization. However, increased temperatures lead to cold-inducible protein degradation. Subsequent temperature decrease then leads to significantly lower *de novo* accumulation of cold-inducible proteins in vernalized winter cereals with respect to unvernalized ones.

Therefore, in regions with mild winters characterized by freeze-thaw periods leading to cold acclimation followed by deacclimation processes, accumulation of cold-responsive proteins such as dehydrins corresponds to plant acquired frost tolerance only in non-vernalized plants. Thus, to use accumulation of dehydrin proteins as an indirect marker of frost tolerance in winter cereals (wheat, barley), sampling of winter-type cereals prior to vernalization fulfillment has to be recommended in regions with temperate climates such as Czechia.

Moreover, given the complexity of the field conditions, changes in photoperiod during winter also have to be taken into account. Early winter samplings at November and December were taken at short-day conditions (shortening of daylength until winter solstice) while January samplings were taken at relatively prolonged daylengths which lead to induction of *FT1/VRN3* gene upstream of the key vernalization gene *VRN1*. A positive effect of prolonged daylength on barley shoot apex development (double-ridge stage) and a decrease in *Cor/Lea* genes and frost tolerance was reported by Fowler et al. (2001) under controlled conditions. Thus, the observed alterations in barley shoot apex development and alterations in DHN5 levels during winter also correspond to daylength prolongation in January when compared to earlier sampling dates in November and December.

## Conclusion

Our study shows for the first time that dehydrin proteins and transcripts can be used as a marker for assessment of plant WS in winter cereals grown under field conditions, but only winter wheat and barley plants prior to vernalization fulfillment have to be used for dehydrin determination. Thus, a positive correlation between WS and dehydrin protein relative accumulation (WCS120 dehydrins in winter wheats and DHN5 protein in winter barleys) was found in winter wheats and barleys in vegetative stage (prior to vernalization), respectively, grown in the field in Czechia.

## Author Contributions

PV outlined the experiments, provided dehydrin analyses, and sampled and interpreted data from all participants. IP and JM provided determination of wheat and barley winter survival. PM provided samplings of winter barley cultivars at location Lužany. LH and PS provided transcriptomic analyses of wheat dehydrins. KK, PV, MK, and IP wrote the manuscript.

## Conflict of Interest Statement

The authors declare that the research was conducted in the absence of any commercial or financial relationships that could be construed as a potential conflict of interest.
