# Supplementary material for: Relationship Between Dehydrin Accumulation and Winter Survival in Winter Wheat and Barley Grown in the Field
Source: Front Plant Sci. 2019 Jan 29;10:7. doi: 10.3389/fpls.2019.00007 (PMC6361858; doi:10.3389/fpls.2019.00007)
Supplement: Supplementary file 1 [file Data_Sheet_1.docx]

**Tab S1**. Lethal temperature (LT50) of wheat and barley varieties sampled from field at Crop Research Institute in 5.12.2013

**Barley LT50 (°C) Wheat LT50 (°C)**

Lester -15.6 Bohemia -20.3

Fridericus -15.1 Dulina -15.2

Lancelot -14.0 Sultan -14.2

Saffron -13.3 Aranka -8.6

**Table S2.** Correlation coefficient of each samplings and some of their averages and winter survival index (WSI). Number of samples is in bracket. Bold number means significant correlation (P ≤ 0.05). Dec –December samplings, Jan –January samplings, Nov –November samplings. Numbers 13 or 14 after Nov, Dec or Jan means year of the sampling. Abbreviation of months without number means average value of accumulation of dehydrins in the cultivars sampled in the same months during both winters. L - field in SELGEN - Lužany, Sum – correlation of all normalized values of dehydrin accumulation in the same genotype obtained during experiment, V - field in Crop Research Institute.

| Barley | Dec13-V (4) | Jan14-V (5) | Nov14-V (5) | Jan14-L (7) | Dec14-L (8) | Dec (8) | Jan (7) | Sum (9) |
| --- | --- | --- | --- | --- | --- | --- | --- | --- |
| WSI | 0.184 | -0.300 | **0.974** | 0.120 | 0.624 | 0.551 | 0.052 | 0.402 |
|  |  |  |  |  |  |  |  |  |
| Wheat | Dec13 (4) | Jan14 (4) | Nov14 (5) | Jan15 (8) | Jan (11) | Sum (11) |  |  |
| WSI | 0.862 | 0.743 | **0.932** | 0.659 | 0.372 | **0.832** |  |  |


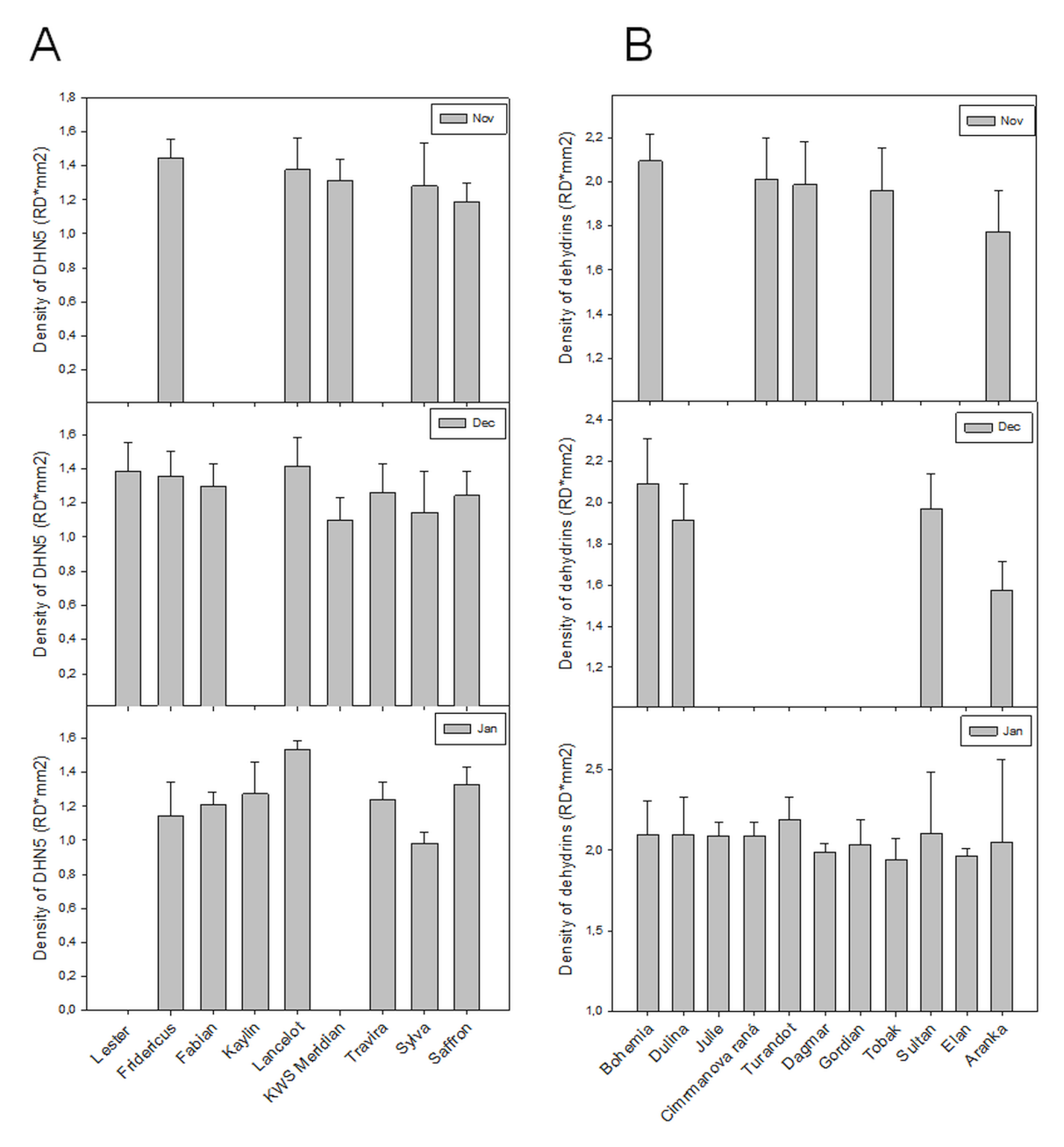


Figure S1. Accumulation of dehydrin proteins in crowns: A) DHN5 in barley and B) total amount of all the most abundant dehydrins (WCS200, WCS180, WCS66, WCS120, WCS40) in wheat. The data come from the immunoblots. Error bars indicate standard deviation (SD). Dec –December samplings, Jan –January samplings, Nov –November samplings.


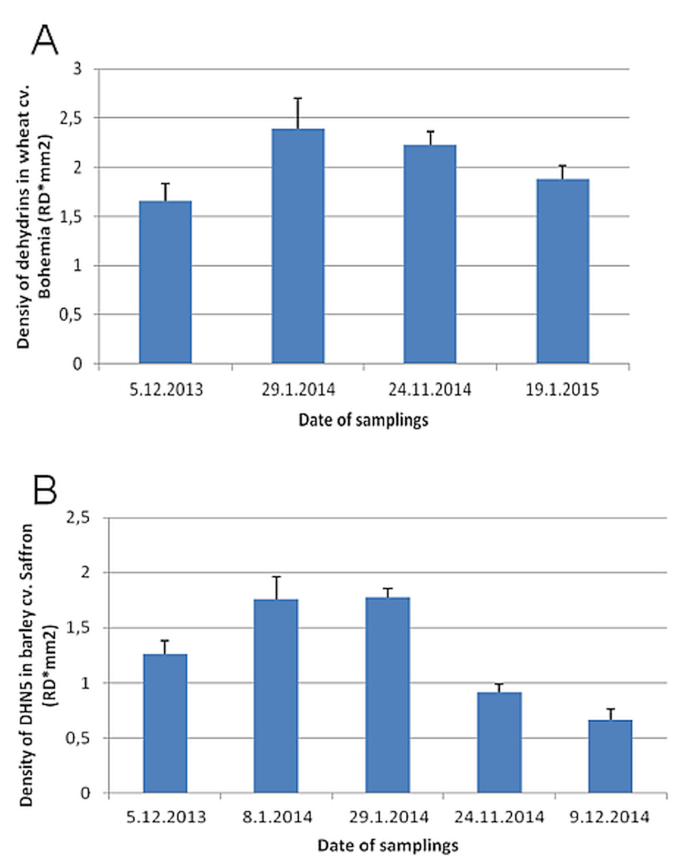


Figure S2. Detailed view on non-normalized values of dehydrins in A) winter wheat Bohemia and B) winter barley Saffron in all samplings. The data come from the immunoblots. Error bars indicate standard deviation (SD).


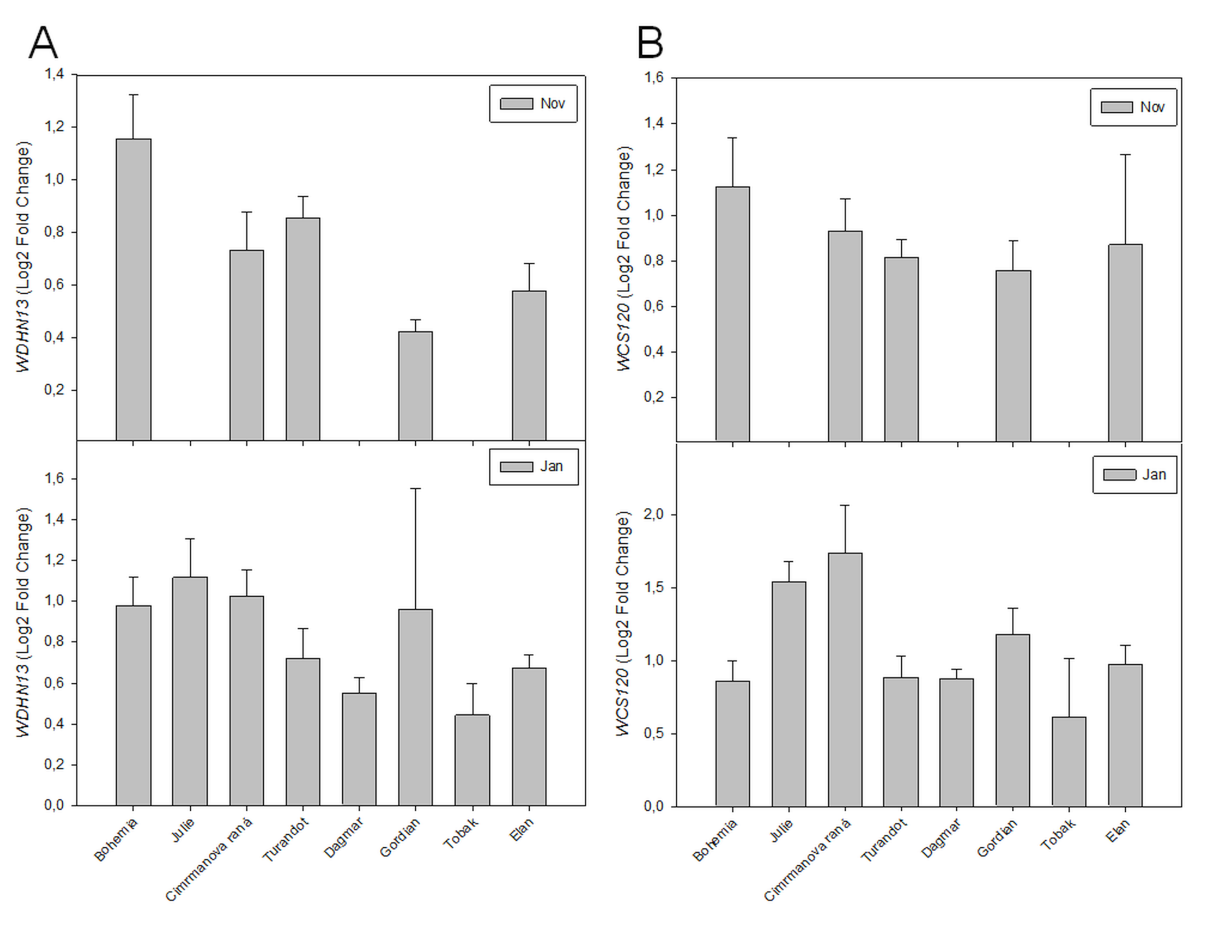


Figure S3. Normalized relative gene expression of dehydrin genes in wheat crowns: A) *WDHN13*, B) *WCS120*. Error bars indicate standard deviation (SD). Jan –January samplings, Nov –November samplings.
